# Can learning organizations survive in the newer NHS?

**DOI:** 10.1186/1748-5908-1-27

**Published:** 2006-10-30

**Authors:** Rod Sheaff, David Pilgrim

**Affiliations:** 1 Health Service Research, University of Plymouth, Plymouth, UK; 2 Lancashire School of Health and Postgraduate Medicine, University of Central Lancashire, Lancashire, UK; 3 Department of Primary Care, University of Liverpool, Liverpool, UK

## Abstract

**Background:**

This paper outlines the principal characteristics of a learning organisation and the organisational features that define it. It then compares these features with the organisational conditions that currently obtain, or are being created, within the British NHS. The contradictory development of recent British health policy, resulting in the NHS becoming *both *more marketised *and *more bureaucratised has correspondingly ambiguous implications for attempts to implement a 'learning organisation' model.

**Methods:**

Texts that define and debate the characteristics of a learning organisation were found by snowballing references from the founding learning organisation books and published papers, and then by searching a database specifically devised for a literature review on organisational structures and processes in health care. COPAC and ABI-Info databases for subsequent peer-reviewed publications that also appeared relevant to the present study were searched.

**Results:**

The outcomes of the above search are summarised and mapped onto the current constituent organisations of the NHS to identify the extent to which they achieve or approximate to a learning organisation status.

**Conclusion:**

Because of the complexity of the NHS and the contradictory processes of marketisation and bureaucratisation characterising it, it cannot, as a whole system, become a learning organisation. However, it is possible that its constituent organisations may achieve this status to varying degrees. Constraints upon NHS managers to speak their minds freely place an ultimate limit on learning organisation development. This limitation suggests that current British health service policy encourages organisational learning-but not too openly and not too much.

## Background

### Modernisation and learning

In 1998 the British Secretary of State for Health announced that a central aim of the incoming Labour government was to 'modernise' the NHS. According to the Secretary of State for Health, this modernisation included the need to:

'...create a culture in the NHS which celebrates and encourages success and innovation...a culture which recognises...scope for acknowledging and learning from past mistakes' [1]

A key plank of this emphasis on learning and innovation was the introduction of a policy of clinical governance [2,3]. The policy emphasised the multi-disciplinary responsibility of colleagues working together in a clinical area to manage risk, implement evidence-based practice, and learn from errors. This quality assurance ethos, in which all staff were encouraged to participate, seemingly indicated that the government wanted to frame service improvements in systemic terms rather than emphasising individual performance alone.

With the above starting point in mind, Davies and Nutley [4] elaborated a relevant organisational development concept, which was already well-known in managerial studies [5], of a learning organisation. In their paper, they set out some aspirations for, and cautions about 'developing learning organisations in the new NHS.'

A few years on, how does this policy intention look, especially given that the 'New' NHS is even newer-more reformed, more 'modernised' ? Our aim here is not to query the descriptions, aspirations, or normative premises set out by Davies and Nutley. Instead, their reflection of the late 1990s period will be placed in the context of more recent health policy and the changed character of the NHS. Our aim in so doing is to interrogate the capacity of recent NHS 'modernisation' activities to realise the earlier rhetoric about enabling its constituent organisations to develop into learning organisations.

For the learning organisation aspiration hinted at by the Secretary of State in 1998 did not exist in isolation from the broader and multifaceted notion of 'modernisation.' It was part of a complex policy weave, containing strands that have been separate from, and apparently sometimes in opposition to, a learning organisation imperative. Elaborating on the scene-setting of Davies and Nutley, we briefly set out, for readers new to the topic, key points about what Senge and other management writers take a learning organisation to be [6]. Then we compare these management theory accounts with recent developments in health policy and NHS management. By doing so we explore how far these developments have established the necessary conditions for learning organisations to develop.

## Methods

Thus, the present method is a criterion-based evaluation. As the criteria by which to evaluate how far NHS organisations have become more like the learning-organisation model, we first identify what organisational norms proponents of the Learning Organisation are broadly advocating. How does a learning organisation differ from other organisations? What peculiar outcomes does it aspire to produce compared to other organisations? How does it produce these outcomes? We found these texts by snowballing references from the founding learning organisation books and published papers, and then by searching a database specifically devised for a literature review on organisational structures and processes in health care [7]. To update this, we also searched COPAC and ABI-Info databases for subsequent peer-reviewed publications that also appeared relevant to the present study. The search terms were learning organisation/organization combined with at least one of: 'health,' 'hospital,' 'clinic,' 'surgery,' 'ward,' 'emergency,' 'NHS,' 'general practice,' 'physician,' or 'provider' in the title, abstract or keywords.

Collectively, these texts elaborate the idea of a learning organisation. *Inter alia *they state the conditions which, they argue, are necessary and sufficient for a learning organisation to exist and achieve its objectives. There is little consensus about the underlying disciplinary bases, conceptual frameworks, learning theories, what is learnt, by whom, and how precisely the relevant learning is institutionalised [8,9].

To sidestep these debates and to avoid the dangers of anthropomorphising organisations [10,11] or treating learning as a variable or 'quasi-object' [9], we assume that organisational learning involves, at minimum, learning by at least some individual organisation members *and *a set of organisational learning mechanisms (structures and processes) that promote their collective action on the basis of that learning – and in pursuit of the organisation's current goals [11,41,12]. On these two points, there is greater consensus. We continued reading through these works until saturation, in the sense that further reading added little to our list of these defining features as characterised by advocates of the learning organisation.

Critics of the idea of a learning organisation also were revealed by this method. Some critics argue that the idea of a learning organisation is desirable but hard to implement in the face of managerial reluctance to share power [13,14]. Others regard learning organisation practices as a tactic for channelling employees' critical and inventive capacities away from resistance to management into the service of the firm [15-17]. Some critics even dismiss 'organisational learning' as part of the wider, and in their view equally specious notion of the 'knowledge economy' [18].

Selecting and reading in a similar way, our second step was to assemble a list of the main criticisms of the accounts of a learning organisation. The main locus of disagreement between critics and advocates is less about what organisational characteristics and outcomes would constitute a learning organisation, but rather about what environmental conditions, organisational structures and processes, if any, are also sufficient to produce the outcomes attributed to learning organisations. On this basis, our next step was to narrow down our list of the defining attributes of a learning organisation to those upon which advocates and critics mostly agree.

Then we compared the effects of recent NHS 'modernisation' activities with that list. The corresponding empirical description of these effects is drawn from secondary empirical research, policy documents, and the authors' own first-hand research and other observations during the period following the debut of the idea of learning organisations in NHS policy [1]. These sources are selected for relevance to the necessary conditions elicited at the third step of the analysis. Published empirical research about learning organisations is meagre compared with the amount of ink spilt in generalities on the subject [19].

A learning organisation is accomplished, its proponents argue, through an *intra*-organisational cultural shift. In competitive markets, a strategic investment in a learning organisation (a cost of time and money) is designed to make the competing company more robust and profitable in the face of less educated and reflective competitors, thus generating an outweighing benefit. There is a clear contrast between single firms, where a 'learning organisation' model can apply, and a whole-market level, where it cannot. This crucial distinction is important to make in the light of the marketisation of the NHS.

We return to this point later, but here we note that since 1998 it has become increasingly simplistic to assume that the NHS can be treated as one whole organisation. However, it is conceivable that within the NHS some of its constituent organisations (e.g., a local general practice, treatment centre or hospital) could develop a learning organisation approach to maintain or increase its competitiveness. So we distinguish three levels of analysis [9]:

1. The whole NHS, a system of many organisations.

2. Each discrete NHS organisation (NHS trust, general practice, primary care trust, health authority etc.) within the system.

3. Individual learning, which is a component, but not the whole, of organisational learning [20].

The present analysis focuses on level 2; that is, on how learning occurs within NHS organisations. Level 1 receives attention only insofar as their external 'environment,' in particular NHS re-structuring, influences whether NHS organisations can be learning organisations. Similarly, individual learning (level 3 above) is considered only insofar it is a corollary of NHS organisations (i.e. entities at level 2) becoming learning organisations. Level 2 has, in organisations of any size, its own internal gradations. A critical question is how far policy changes – here attempts to implement learning organisation norms in large NHS organisations – penetrate 'down' each organisation from senior management to the actual delivery of clinical care. We focus not on the whole concept of 'organisational learning' (empirical accounts of how organisations learn), but on the narrower concept of a learning organisation, such as a normative model of organisational structures and process, whose empirical, but not evaluative, elements may be evidence-based.

The present method has the advantage of taking into account the views of both supporters and sceptics about learning organisations. The corresponding limitation is that we accept the consensus between them as a working assumption rather than expose it to empirical testing. We acknowledge that future research may show that we have conceded too much in doing so. Furthermore, the values which a criterion-based evaluation applies are always open to debate. It has been argued that the climate in learning organisations is not 'utopian sunshine,' but 'Foucauldian gloom' [21].

### Defining features of a learning organisation

Weber used the term 'ideal type' to describe model forms of organisation. In the case of a learning organisation, the seminal text describing the desirable 'ideal type' is offered by Senge [6]. Some organisational researchers, in particular Snell [35], have compared Senge's ideal type features against attainable best practice. Senge considers that a learning organisation should not only aspire to, but also *achieve *his five ideal type features (the 'defining features' listed below). As Snell notes, this would require a super-human effort for any organisation no matter how culturally secure and financially well-resourced. Snell offers some less utopian practical guidance from the learning organisation literature. It does not contradict Senge, but it is less conceptual, more descriptive and pragmatic.

#### Competence and ways of thinking

Models of learning organisations are mainly derived from studies of the more adaptive commercial firms [22,23], though not exclusively [24]. As noted, one requirement of a learning organisation is that at least some of the individuals within it learn how to work more effectively. A learning organisation thus involves:

1. *Maximising individual competency: *Improvements in consumers' experience or other working practices can only be achieved if the workforce is well educated and that education is constantly refreshed. This requires the organisation supporting each individual to make the best of their aptitudes and abilities in the above directions, and to build on them continuously ('life long learning'). It also requires that most of the individual members of an organisation work in the ways listed below, especially the 'leaders' [25,26]. However, a set of competent individuals does not a learning organisation make. Further, particular organisational conditions also are said to be necessary, beginning with the following specific shared ways of thinking.

2. *Open systems thinking *entails people in learning organisations, especially those in leading positions, seeing the bigger, environmental picture and where they and their particular functional or physical setting fit in to that picture [27]. In particular, they need to see two aspects of their organisation's external environment: the emergence and activities of competitors or substitutes for their own activity, and the emergence of new technologies for undertaking that activity; in short, learning 'beyond the walls' [28]. The opposite of this is thinking within the closed bureaucratic, parochial or professional world of their existing activities.

3. *Team learning *is important whenever tasks are delivered in teams – a team being all those people of different occupations who are collectively engaged in producing one of the organisation's products or services. A learning organisation attempts to formalise the tacit knowledge that production teams rely on [25]. For NHS organisations that would imply that 'modernisation' policies have actually impacted on the teams that deliver clinical care and, if so, promoted rather than impeded team learning.

4. *Updating 'mental models' *entails people in learning organisations understanding their own assumptions about their work and appreciating their colleagues' assumptions. Team learning and open systems thinking depend upon each person understanding the mental models they hold themselves, and understanding and appreciating those which others hold [29,30], so that members of different occupations repose increasing trust in one another. A concomitant is a capacity for 'unlearning' obsolete or counter-productive mental models [31].

5. *Cohesive vision *refers to clarity of unifying purpose in an organisation [32] and 'guiding ideas' about strategies to achieve it [33]. Learning organisations develop ways of owning a shared vision throughout the workforce. As a result, members of different occupational groups trust higher management. This cohesive vision could emerge from the bottom but is usually engendered from above. A cohesive vision is one important dimension to developing a learning organisation, typically engendered by good leadership. For this reason, leadership that champions learning and puts it at the centre of organisational functioning is vital to developing a proper learning organisation.

#### Organisational culture

A concomitant of most organisation members working in the ways described above is that the official culture of the organisation changes accordingly; it becomes a *learning culture*. Employees would accept the need to be flexible and adaptable. Reciprocally, employers would demonstrate a clear commitment to continued professional development. Mintzberg et al, [34] suggest which cultural processes typify learning organisations. They say that learning organisations: celebrate success, avoid complacency, tolerate mistakes, believe in human potential, recognise and value tacit knowledge and respect work based competence, are open to diverse and flexible ways of sharing knowledge and experience, and engender trust, horizontally as well as vertically in the organisation. Finally, learning cultures should be outward-looking not insular. Other writers propose their own catalogues of 'organizational learning values' [11]. Snell [35] therefore suggests that learning organisations would show clear empirical signs of:

1. *A community of learners: *In general, the membership of a learning organisation would show signs of goodwill, solidarity and collaboration with their colleagues. It would be inclusive, incorporating all ranks and professions [13]. It would place a premium on the validity of information and knowledge [11].

2. *Learning leadership is dispersed *throughout the organisation. From situation to situation, individuals would move readily between the roles of learner, co-learner, coach, pupil, mentor or teacher. A formalised, top-down hierarchy with fixed roles is inimical to this kind of learning [14] – a flexible non-defensive culture that is open to experience and opportunities for learning and whose participants recognise that expertise is distributed amongst them [36].

3. People are confident to have an *open dialogue *about multiple perspectives [13]. Uncertainty and contested viewpoints would be clearly tolerated. People would not be fearful of speaking their mind, of expressing doubts or exposing mistakes, of critical thinking, or of using knowledge from outside the organisation [37,20].

4. *Ongoing collective transformation *and self-improvement are evident, in particular changed working practices [38,41] and the corresponding 'theories in use' [39]. One sign of this is that working processes are 're-engineered' [40] rather than changed in relatively superficial ways [13]. Organisation members' 'theories-in-use' also would change [39], and not all change is the result of learning [41,9].

All the above conditions involve a degree of trust between different occupational groups. Trust, a feature of a learning culture, takes time to develop. Organisational structures that are too short-lived engender distrust, a point that Sennett [42] emphasised in his critique of transferring the principles of an unstable, rapid turn-over business culture to state bureaucracies. Learning organisations are expected to be open to change, but too much change brings with it a lack of trust. What happens then is not cultural change but culture shock, which is disabling because it produces personal defensiveness and resistance.

#### Triple learning

Using NHS examples, Davies and Nutley [4] define three types of learning. 'Single-loop learning' entails an audit identifying the gap between intended and identified performance and installing corrective action. In 'double-loop learning' wider lessons are learned about organisational performance from audits and evaluations and larger adjustments are made at the level of organisational goals and direction, with implications for organisational structures and working practices [43]. There is a transfer of learning from an example to one or more others. Third, there is 'learning about learning'. This entails people in learning organisations taking stock, not just of the content of organisational lessons but the process by which this learning took place [37] – a form of reflexivity for the betterment of the organisation. Learning organisations would achieve this higher order type of learning or 'meta-learning,' not just accumulate single- and double-loop lessons.

#### Dynamic capability and knowledge management

Proponents of the learning organisation maintain that the cultural shifts noted above provide organisations with advantages. Productivity is increased and, because of the emphasis on being outward looking and on whole systems sensibility, organisational adaptability is improved. Creative adaptation or 'dynamic capability' arises from the genuine rather than rhetorical enactment of learning organisation principles, in the presence of other enabling organisational features noted below [44].

A genuine internal commitment to a learning organisation approach is a necessary but not a sufficient condition for developing dynamic capability. For an organisation to ensure dynamic capability, first it must become a learning organisation in practice, and second it must be confident and opportunistic about applying what it has learned. Team members need to have trust in one another and enjoy the managerial mandate to exploit opportunities as they arise, or experiment with new conditions emerging from the shifting external context that situates the organisation.

Thus, the rhetoric of a learning organisation can be tested on a case-by-case basis (as we do below in regard to English NHS organisations) against what the organisation actually practices. For example, the ill-fated Rover automobile company claimed to be a learning organisation but only established one main feature (maximising the individual learning of its workforce) [45]. By contrast, Chaparral Steel in the USA, a more stable and successful company in the 1990s, reportedly demonstrated its learning organisation credentials and accrued the benefits of dynamic capability [46]. Such claims also are made for BP [47] and, in more guarded terms, for other firms [48]. A critical difference between these companies was that Rover outsourced its attempt at becoming a learning organisation, whereas the other two developed it from their own senior managers. The latter championed and oversaw fidelity to the learning organisation model as a corporate rather than a brought-in managerial initiative. We return to the importance of leadership in a learning organisation later.

Research and development are one aspect of a learning culture. Successful knowledge management, a concomitant or implication of a learning organisation, also is said to increase dynamic capability [49]. Ownership of intellectual property is a commercial advantage in itself, as is the capacity to deny that knowledge to competitors, but its main use is the utilisation of knowledge to achieve an organisation's operational goals and strategic aims. The most obvious example of this is knowledge-based decision-making at all levels in an organisation. (The existence of this very journal testifies to the logic discussed here.)

It is generally assumed that the creation of learning organisations requires the combination of all the conditions listed above, not just some of them.

### From 1998 to 2006: Can the current NHS nurture learning organisations?

The foregoing lists only the main conditions required for a learning organisation. It highlights the role that a learning organisation approach could play in raising clinical quality and NHS efficiency. To what extent has NHS 'modernisation' tended to create each of the afore-listed conditions to enable its constituent organisations to emerge as learning organisations?

#### Open systems thinking and the updating of 'mental models'

These activities have become more prevalent activities in NHS organisations since 1998, as part of a complex and sometimes contradictory policy weave. It has included policies promoting: research and development, improving the patient experience, risk-management, deliberate structural destabilisation, and workforce development and leadership. In regard to health policy and management, NHS organisations have in some cases been strongly encouraged to update their mental models, in particular to examine, even adopt, working practices and models of care (e.g. the Kaiser Permanente [50] and Evercare models [51] that appear to have proved valuable in other health systems, especially that of the USA). The links between health policy and NHS management targets, tasks, and imperatives, on the one hand, and national policy agendas, on the other, have become increasingly salient and transparent. Against this trend, Vassalou [20] describes some NHS managers' limitations in thinking 'outside the walls' of existing practice.

#### Team learning

The sort of team learning that learning organisational theorists advocate runs against the grain of meritocratic educational structures from which a clinical professional typically comes into the workplace [52]. Those structures emphasise individual learning and scholastic achievement – not collective learning. Clinical activity develops its own self-sustaining logic, which tends to displace protected learning time because of the opportunity costs involved and the risks accruing to activity targets. In the case of independent practitioners, these are direct financial costs and thus very powerful disincentives. The only learning that might be guaranteed comes from uni-disciplinary, individualised and defensive requirements for appraisal, clinical supervision and the enlarged stick (in the UK post-Shipman) of professional re-validation [53].

NHS management also relies on heavily top-down information flows, whilst at the same time attempting to involve clinicians ever more closely in management [20]. There also are reports that NHS nurses and managers remain deferential to, even cowed by, senior hospital consultants and of a still deep-rooted NHS culture of knowledge flowing from doctors to other professions [54]. Within parts of the medical profession itself, there is evidence of the threat of managerial interference being used as a means of 'soft coercion' in the management of clinical governance [55]. These tendencies are antithetical to a learning organisation [56], which, as explained above, is intended to be non-defensive, multi-disciplinary, and characterised by team and not only individualised learning.

#### Cohesive vision

Improvements in the patient experience have remained at the top of the political agenda and managerial targets, and these improvements are defined primarily in terms of access to services (e.g., waiting times, choice and variety of providers). In late 2005, a renewed focus on financial targets was added. In terms of policy targets, since 1998 the NHS has had a highly cohesive vision. But for its organisational structures, the term 'policy mess' comes to mind. The frequency of successive major structural reforms to the NHS is accelerating. In more recent times, particular initiatives have been announced with gusto one moment only to be very quietly dropped the next. The House of Commons' Health Committee, for one, has criticised policy towards Primary Care Trusts (PCTs) for its zigzags and apparently being made up by decision-makers as they go along [57].

There are other examples: reforms in 2006 have reduced PCT numbers dramatically and effectively shifted the reduced Strategic Health Authority configuration back to the older pattern of large Regional Health Authorities. GP fund-holding was first abolished then essentially reintroduced under a new name (practice-based commissioning). These events are not symptoms of a coherent health policy vision for the NHS or its constituent organisations. Since 1998, ministers have promoted the provision of services by non-NHS, especially commercial, providers and the diversification of organisational variants of NHS providers [71]. Indeed, government ministers have taken pride in boasting this intention about destabilisation, with the paradoxical injunction that instability is a form of strategic coherence.

The commissioning and provision of services are to be increasingly separated, and so another systemic tension has been deliberately introduced. Competition is encouraged among providers and international competitors are solicited. Intentionally or not, a policy of provider 'contestability' suggests to many local health care professionals not that they are trusted and valued, but that they are dispensable. Then, the creation of one condition (i.e. competition) stimulating the learning organisation approach negates another condition (i.e. trust between professionals and management).

Another lack of cohesion appears in regard to models of leadership. In the past five years 'leadership' in the NHS has been encouraged by politicians and civil servants. Potentially this is another driver that could encourage a learning organisation approach, but a great deal depends on what policy-makers mean by 'leadership' and what they regard as their 'ideal type' of leadership.

For example, the Banff Centre for Creative Leadership emphasises action learning. It utilises Kolb's experiential learning cycle (concrete experience followed by reflection followed by abstract conceptualisation followed by active experimentation leading to a new concrete experience) [58]. This learning cycle captures the dynamic logic of the cultural features noted earlier of a learning organisation [34]. The leader of a learning organisation would necessarily manifest a mixture of consistent vision and personal humility. This model of leadership comes close to 'learning organisation' norms [8].

A very different model is the 'boot camp' type developed by Tichy at the University of Michigan Business School [71]. In this approach to leadership, aspiring leaders go on energetic and demanding courses where they have to become role models for their workforce. They must be stretched in their ambitions and their commitment to work, in their focused imagination and their devoted time and energy. Participants have to work intensively for long hours on projects, and then they receive elaborate critical feedback about their performance. At times, NHS managerial practice displays a similar approach to leadership, with managers, and especially chief executives, facing strict targets with strong personal penalties for failing to meet them, reinforced by investigative and, occasionally, punitive methods for 'helping' NHS trusts in financial difficulties.

This emphasis on strong decisive decision-making at the top is thus one brand of leadership, culturally reinforced in the recent British context by TV programmes like *The Apprentice *lead by the bullish Alan Sugar. This model of leadership encourages individual charisma or even authoritarianism. There is some evidence [60] that this model is being politically preferred in the NHS as the vehicle for prompt, single-minded implementation of the targets mentioned above. If this is the case, it is a form of leadership at odds with that implied in the learning organisation literature.

#### Maximising individual competency

As noted, learning in the clinical professions has tended to be uni-disciplinary and individualised. These arrangements make for strong individual competency rather than the non-defensive, multi-disciplinary team learning that a learning organisation is said to require. However, even individualised learning has had recent vicissitudes in the NHS.

The first 'Wanless Review' [61] assumed that the NHS should spend 10% of its resources on quality improvement through learning (of all kinds) by 2010, a substantial rise from between 2–5% in the 2002 baseline estimate. It has become a standard requirement of every NHS professional to prepare and implement an annual Professional Development Programme (PDP), and in many localities clinical facilitators have been appointed to assist this activity. Individual learning takes time, which incurs opportunity costs, and clinical and managerial duties must be covered when learning events occur ('backfill' is needed). In a cash-strapped system it is easy for learning to be demoted in importance or become a casualty of the most recent round of cost-savings demanded to balance annual budgets.

Since 2005, financial performance indicators have become more stringently applied, rendering protected learning time more vulnerable. For many NHS staff, a combination of increasing work loads and central control reduces their practical scope for experimentation [20]. Financial retrenchment and uni-professional defensiveness, in the face of politically elicited culture shock, undermine the support for the organisational shifts and risks attending the development of a learning organisation.

Despite the continuing emphasis on the '3Rs' (see below) year-on-year cash deficits are now leading some parts of the NHS to shed rather than recruit staff. Education, training and re-training have been among the first financial casualties of the stricter NHS financial regime of 2005–6. This component of a learning culture in the NHS would only be possible if adequate money for learning and development was consistently guaranteed. The opposite is occurring at present. With structural change and systemic turbulence washing over the clinical workforce and shorter-term goals being frenetically pursued by NHS managers, the nurturing of a learning organisation approach and culture is easily pushed down the order of organisational priorities.

#### Negotiating cultural change

Increased bureaucratic complexity and the weakening of professional authority have been features of NHS life in the past few years. These are aspects of a narrowing conception of accountability that increasingly focuses on compliance with targets and risk avoidance. Besides clinical governance itself (see below), another example here is the Research Governance Framework installed in reaction to scandals involving poor informed consent for clinical research at hospitals in Bristol, North Stafford, and Liverpool (Alder Hey). During the same period, the Shipman Inquiry into a general practitioner who murdered many of his patients put forward recommendations to control poorly performing doctors and reduce risk in primary care. These events have now rendered clinical professionals as perennially suspect social actors. Trust in a professional ethos has been displaced by a more distrusting political attitude. Horizontal bonds of goodwill and trust are being replaced by more and more systems of upward vertical accountability, which increase rather than decrease the probability of a blame culture.

Taken with systemic turbulence, this vertical emphasis means that management cultures are often short-lived, and their leaders may be disposed of if short-term goals are not achieved. They are only as good as their most recent local delivery plan or star rating attainment [54,60,62]. As a consequence, a unifying intra-organisational culture has not been fostered. Instead, the NHS has been fragmented and sub-systems and interest groups have been set against each other. This is not a propitious starting point to develop a cohesive, mutually trusting, honest and reflective culture with a common unifying vision. In a learning organisation, the ethos of 'horizontal' team learning emphasises knowing thyself – and thy colleagues. In a culture where vertical one-way accountability predominates, the emphasis instead is on knowing thy place.

#### Community of learners

Workforce development has always been an important aim of the NHS, but recently it has become more so. The NHS has large labour shortages in many areas and the '3Rs' (recruitment, retention, returners) tax the minds of its managers. Some localities cannot attract health workers, and there are not enough of them overall. To make the NHS an attractive and reliable employer, the personal development of individual staff is now encouraged by appraisal systems and frames of external reference such as *Improving Working Lives *.. In its design the NHS *Knowledge and Skills Framework *moves away from a 'silo' conception of self-contained bodies of knowledge, each particular to one profession, toward the idea of a core body of clinical expertise shared by all professions, but elaborated into different specialties and to different degrees of depth by different occupational groups.

Alongside, a relaxation of inter-professional demarcations (in particular, the shifting demarcations between nurse practitioners, physician assistants, and general practitioners) points toward the more flexible, adaptive workforce of the learning organisation. These developments fit the idea of a 'community of learners.' Against this, Currie and Suhomlinova [63] record the divergence of clinical and academic medicine due to the policy pressures of NHS targets and the Research Assessment Exercise, respectively, and a still deep-rooted NHS culture of knowledge flowing from doctors to other professions.

#### Dispersed learning leadership

The success of clinical governance has been defined negatively by the absence of adverse incidents and positively, but very narrowly, by persuasive annual reports to NHS Trust Boards from a small named sub-system (the 'clinical governance department' or its equivalent), as well as its responsible, and so potentially blameworthy, Executive Director. What started as a rallying call about collective team responsibility for quality at the clinical 'coal face' has turned into standard setting focussed on performance indicators, the application of policies and procedures, and forms of bureaucratised vertical accountability. This move toward bureaucratisation has been described in general practice [64] beside hospital medicine.

The learning organisation discourse of dynamic bottom-up 'clinical governance' has gradually elided towards a static and codified top-down one of 'health standards.' The original aspiration of clinical governance being a bottom-up, collectively-owned responsibility for clinical quality was completely consistent with developing a learning organisation ethos. However, with the pressure for vertical accountability (see above) rather than horizontal trust and team commitment to service quality, clinical governance has been transformed in the past few years into a narrow devolved responsibility for one sub-system of clinical care, not for the whole system as originally intended.

The research governance framework (RGF) was introduced at a time when a variety of capacity building exercises in the NHS had been designed to encourage more research and development in the clinical workforce. However, the RGF has become a defensive and bureaucratic process. It may perhaps, although there is precious little evidence either way, be lowering the risk to patients of sub-standard research. However, it has certainly had the effect of producing disincentives and obstacles for all researchers, but especially for neophytes. Less, not more grass roots learning is likely as research increasingly becomes the possession of elite university-based departments. The latter are overwhelmingly preoccupied by research not development, driven by non-NHS incentives in higher education such as the Research Assessment Exercise and grant chasing [63]. As a consequence, development, the natural terrain of learning organisation enthusiasts in the NHS, will diminish in organisational importance because it is a burden or dutiful afterthought for academic researchers. This tendency will now increase as local control for the RGF is to be sited in new regional offices and elite academic research is being privileged over service development [65].

#### Open dialogue

The narrowing focus of accountability (see above) has tended to make NHS management past-present focused – testing performance against business or 'delivery' plans and the personalised objectives flowing from them, characterised by vertical accountability and short-term target-achievement. In its most extreme form of hierarchical functioning, pragmatism and short-term interests, it is antithetical to the 'learning organisation' model. The extent to which NHS managers are permitted publicly to discuss clinical or organisational problems of their organisations, and even the forms of words which they are required to use when they do, have become increasingly circumscribed, pre-scripted and formulaic. This approach may make for effective news management but not for the open dialogue which organisational learning is assumed to involve. It stands in particular tension with the principles of evidence-based management.

#### Evidence-based medicine and dynamic capability

In regard to clinical 'technology,' the spread of EBM/EBP has been promoted for that very purpose. The spread during the last decade of evidence-based practice has been a bedrock of NHS clinical governance policy. In the context of the NHS, knowledge management has been partly driven by the evidence-based medicine movement, partly by the move to use IT systems to increase efficiency, and partly by frameworks such as Total Quality Management and other initiatives to re-engineer health systems. The NHS has supported it with a rapidly growing R&D programme, and the NHS knowledge and skills framework described above.

#### Triple-loop learning in the NHS

Risk-management has become a pervasive aspect of the NHS management ethos. To minimise clinical and organisational risks the NHS has been exhorted to become an 'organisation with a memory,' minimising present and future errors by learning from those evident in the past. One aim of clinical governance policy and, in a more formal way, case management in primary care (embodied in community matron policy) is to make the audit of services both at care-group and individual patient level an increasingly routine practice within NHS organisations.

Getting knowledge into practice is a challenge for all of the non-clinical aspects of NHS work, including its management processes. Unless this is overcome, best practice is not ensured and neither clinical nor organisational risks are minimised. Since 1998, the NHS has become particularly and increasingly interested in reviewing its own R&D policy and resourcing – the third component of 'triple loop learning' – and in the D of R&D to overcome the problem of getting research into practice ('GriP'). There also is evidence that clinical governance activities have affected some changes in clinical practice, but more in acute care with its relatively well-specified outcomes and working practices than in socially-oriented areas such as mental health care, where the opposite conditions apply [66-69].

## Discussion: Learning, but not too much

In a prescient text about the prospect of marketisation of the public sector, the political scientist Claus Offe came to the conclusion that Western democratic capitalism cannot live with the welfare state, but also cannot live without it [70]. Margaret Thatcher soon discovered this in the 1980s, and Tony Blair has struggled with his own version of contradiction management since 1997. These policy shifts have produced an accumulation of contradictory organisational effects, making the NHS now *both *more bureaucratised *and *more marketised than in the 1980s. It is neither fish nor fowl.

There is a difference between the organisational and the system levels when it comes to health policymakers trying to introduce the notion of a learning organisation. It seems unlikely that the quasi-market structures that increasingly characterise the NHS could successfully encourage a learning organisation approach NHS-wide. Quasi-market relationships between episodically competing constituent organisations would appear more likely to engender distrust rather than trust, empirically challenging us to identify when and at what level, in complex systems, competition is and is not 'healthy' – the new hope of 'contestability.' Attempts to introduce a learning organisation approach for the NHS, as a whole, seem hard to reconcile with the policy, common to both the Thatcher and the Blair governments, of introducing more market-like organisational structures into the NHS.

However, it might be argued that this is to criticise a 'straw man' policy because applying the term learning organisation to the whole NHS is, after all, a conceptual muddle (see above). This is why we previously distinguished level 1 (the whole NHS) from level 2 (its constituent sub-systems). A learning organisation approach could potentially thrive in a well-funded, unified and politically stable State bureaucracy, as well as in a fully autonomous business in a competitive market, or, indeed, in a single autonomous organisation operating within a competitive but publicly-funded health system (a 'quasi-market'). A more penetrating question is whether at the level of its constituent organisations, conditions in the NHS are equally inhospitable to learning organisations.

At that level, the combination of marketisation and bureaucratisation produces a paradox. On the one hand, current health policy and management priorities include some identifiable positive imperatives that give support to the project of making the constituents of the NHS into learning organisation. The creation of competitive pressures imitates one stimulus, in the commercial world, for organisations to become learning organisations. The NHS has become more explicitly critical in reviewing new working practices and clinical technologies, but by the same token more open to adopting those that do prove to be evidence-based. Recent NHS policies on risk-management, clinical governance, and workforce development include elements that would tend to lead NHS organisations toward becoming learning organisations.

On the other hand, there is the rub in current times: these drivers also confront several powerful contemporary systemic constraints or 'challenges' in the daily lives of NHS clinicians and managers. The same system of accountability, which has mandated new models of care, clinical governance, and evidence-basing also has stimulated the increasingly centralised and authoritarian leadership ('performance management') and the bureaucratisation of clinical governance and research governance within the constituent organisations of the NHS. These changes suggest to many clinical professionals the opposite of trust between government (and therefore NHS management) and themselves.

The capacity of NHS organisations to follow 'learning organisation' norms remains constrained by two powerful interests – policymakers and clinicians. Policymakers are often disinclined to publicise, let alone openly learn from, organisational evidence or experience that challenges current policy norms. We also have pointed out some tensions between learning organisation norms and the institutions through which the clinical professions continue to train and socialise their members. These interests constrain the process of organisational learning in the NHS and, when it challenges policy interests, what substantive lessons may be learned too.

The current working solution to this paradox is that NHS organisations are permitted, nay encouraged to learn, but not too much and not too openly. Narrowly, technical learning is encouraged. However NHS managers – in some respects the people best placed to report on the actual implementation and effects of current health policy implementation at service level – are not usually permitted to comment, other than supportively, about current health policy and the effects of implementing it. This limitation, incidentally, also is reported outside the NHS. Most advocates of the learning organisation, and learning organisations themselves, rarely suggest questioning the organisation's most fundamental goals or managerial regime. Those are taken as a given [24,37] [71].

However, another solution is more consonant with learning organisation norms and not restricted to the health sector. It is to allow public sector managers to speak freely, provided they do so in good faith and with sound evidence, about what they have learnt about the evidential basis of current policy and its effects from local experience of their implementation.
